# A laser parameter study on enhancing proton generation from microtube foil targets

**DOI:** 10.1038/s41598-022-14881-9

**Published:** 2022-06-27

**Authors:** Joseph Strehlow, Joohwan Kim, Mathieu Bailly-Grandvaux, Simon Bolaños, Herbie Smith, Alex Haid, Emmanuel L. Alfonso, Constantin Aniculaesei, Hui Chen, Todd Ditmire, Michael E. Donovan, Stephanie B. Hansen, Bjorn M. Hegelich, Harry S. McLean, Hernan J. Quevedo, Michael M. Spinks, Farhat N. Beg

**Affiliations:** 1 Center for Energy Research, University of California - San Diego, La Jolla, CA 92093 USA; 2 Center for High Energy Density Science, University of Texas, Austin, TX 78712 USA; 3 General Atomics, Inertial Fusion Technologies, San Diego, CA 92121 USA; 4 Lawrence Livermore National Laboratory, Livermore, California 94550 USA; 5 Sandia National Laboratories, Alburquerque, NM 87123 USA

**Keywords:** Plasma-based accelerators, Ultrafast photonics

## Abstract

The interaction of an intense laser with a solid foil target can drive $\sim$ TV/m electric fields, accelerating ions to MeV energies. In this study, we experimentally observe that structured targets can dramatically enhance proton acceleration in the target normal sheath acceleration regime. At the Texas Petawatt Laser facility, we compared proton acceleration from a $1\, {\upmu }\hbox {m}$ flat Ag foil, to a fixed microtube structure 3D printed on the front side of the same foil type. A pulse length (140–450 fs) and intensity ((4–10) $\times 10^{20}$ W/cm$^2$) study found an optimum laser configuration (140 fs, 4 $\times 10^{20}$ W/cm$^2$), in which microtube targets increase the proton cutoff energy by 50% and the yield of highly energetic protons ($>10$ MeV) by a factor of 8$\times$. When the laser intensity reaches $10^{21}$ W/cm$^2$, the prepulse shutters the microtubes with an overcritical plasma, damping their performance. 2D particle-in-cell simulations are performed, with and without the preplasma profile imported, to better understand the coupling of laser energy to the microtube targets. The simulations are in qualitative agreement with the experimental results, and show that the prepulse is necessary to account for when the laser intensity is sufficiently high.

Laser-driven ion accelerators will open up a broad range of applications not yet achievable with conventional radiofrequency accelerators. Their spatial ($\sim \,{\upmu }$m) and temporal ($\sim$ ps) compactness, along with their high current density ($\sim \,10^{10}$ A/cm$^{2}$)^1^, make them an ideal ion source for conducting high-energy density science experiments. Laser-driven proton beams have had great success in the radiography of dense plasmas^2^, as well as generating and probing warm dense matter states^3,4^. The laser-acceleration of heavy-ion beams is of interest as well, for its ability to decrease the size and running cost of heavy-ion accelerators, and for the table-top production of rare isotopes^5^. However, much work needs to be done in order to improve the laser-to-ion conversion efficiency in order for ion beams to achieve their potential for ion fast ignition^6^ and cancer therapy^7^.

The most practically robust mechanism of laser-ion acceleration is known as target normal sheath acceleration (TNSA)^8^. When a laser is incident on a thin foil, a strong ($\sim$TV/m) sheath field $E_{sheath}$ is generated on the rear foil surface, which scales as $eE_{sheath} \sim T_{hot}/ \lambda _D$, where $T_{hot}$ is the hot electron temperature and $\lambda _D$ is the Debye length. TNSA ion energy exhibits a modest intensity scaling, generally as $E_{max} \propto I^{1/2}$ for $\sim$ 0.3–1 ps pulse durations^9,10^, or $E_{max} \propto I$ for ultrashort (10s of fs) pulse durations^11^. To improve the transfer of laser energy to ions, one must first increase the absorption of the laser pulse by electrons. One such avenue is to engineer foil targets with structures in the primary laser interaction region, as opposed to interacting with a simple flat foil. Such structures have recently been measured to absorb over 70% of the laser energy^12^, as opposed to the typical few tens of percent with a flat target^13^.

Structured targets have seen tremendous success in improving the conversion efficiency and temperature for laser-driven electrons, in both PIC simulations and experiments. Such structures include carbon nanotubes^14^, nanowires^15–18^, nanoplates^19^, foams^20^, cones^21,22^, and microtubes^23–26^, all of which are superimposed upon flat foils. Targets with non-protruding structure, such as layered foils^27^ and microchannel slabs^28^ have also been observed to improve hot electron generation. As electrons are the mediator for energy transfer into the target, similar structures are of interest for a wide variety of applications, including neutron generation^29^, X-ray and $\gamma$-ray emission^30,31^, positron generation^32^, and QED studies^33^. However, the focus of this work is the performance of structured targets in enhancing laser-ion acceleration. Experimental studies have been done to optimize the ion energy and yield from nanospheres^34^, nanowires^16,35^, snow targets^36^, layered foils^27^, foams^37,38^, and microtubes^39^. Various microstructures are predicted to improve the general TNSA scaling law from $E_{max} \propto I^{1/2}$ to a more favorable linear scaling, $E_{max} \propto I$^21,40^. This hypothesis has growing support from several experiments, showing $E_{max}$ can even double under the right laser and target conditions. In addition, these experiments show that the proton yield relative to unstructured foils is usually increased by a few hundred percent^16,34^.

A complication of structured targets is that they are not readily available and have to be custom-fabricated. In addition, the laser contrast requirement is quite high for structured targets, demanding techniques such as plasma mirrors or frequency doubling crystals. Even minor prepulses with contrasts of $10^{10}$ have been shown to mitigate the effect of structured targets for ion generation, and accounting for the preplasma has been shown to be critical in ensuring the best match with supporting PIC simulations^35,41^.

Here we extend upon previous microstructure target studies with a laser parameter study on a fixed target geometry. With the ultra-high contrast ($\sim \,10^{12}$) PHELIX laser, operating at $\sim 10^{21}$ W/cm$^2$ with a 500 fs pulse length, only an enhancement in conversion efficiency was measured^39^. With such a long pulse length and high-intensity, the short tube was optically shuttered before it could perform optimally, behaving similar to a flat foil with a preplasma. Here we present a comparison of flat vs microtube targets under 3 laser cases: (I) 450 fs, $I = 4 \times 10^{20}$ W/cm$^2$, 82 J; (II) 140 fs, $I = 4 \times 10^{20}$ W/cm$^2$, 28 J; and (III) 140 fs, $I = 1 \times 10^{21}$ W/cm$^2$, 82 J. A direct comparison with fixed energy, pulse length, and intensity reveals that the full energy configurations, I and III, show no benefit of using the microtube targets over flat foils. However, Configuration II accelerates more than 8 times as many protons beyond 10 MeV, with a 50% increase in proton cutoff energy. A 12% improvement in hot electron temperature is also observed.

## Experimental setup

**Table 1:** The Texas Petawatt laser was tuned to three different configurations.

Config.	Intensity (W/cm$^2$)	Energy (J)	Pulse duration (fs)
I	$4 \times 10^{20}$	82	450
II	$4 \times 10^{20}$	28	140
III	$1 \times 10^{21}$	82	140

The parameters were carefully chosen, within the capabilities of the facility, to make a direct comparison of target performance as a function of laser intensity, energy, and pulse duration.

**Figure 1:**
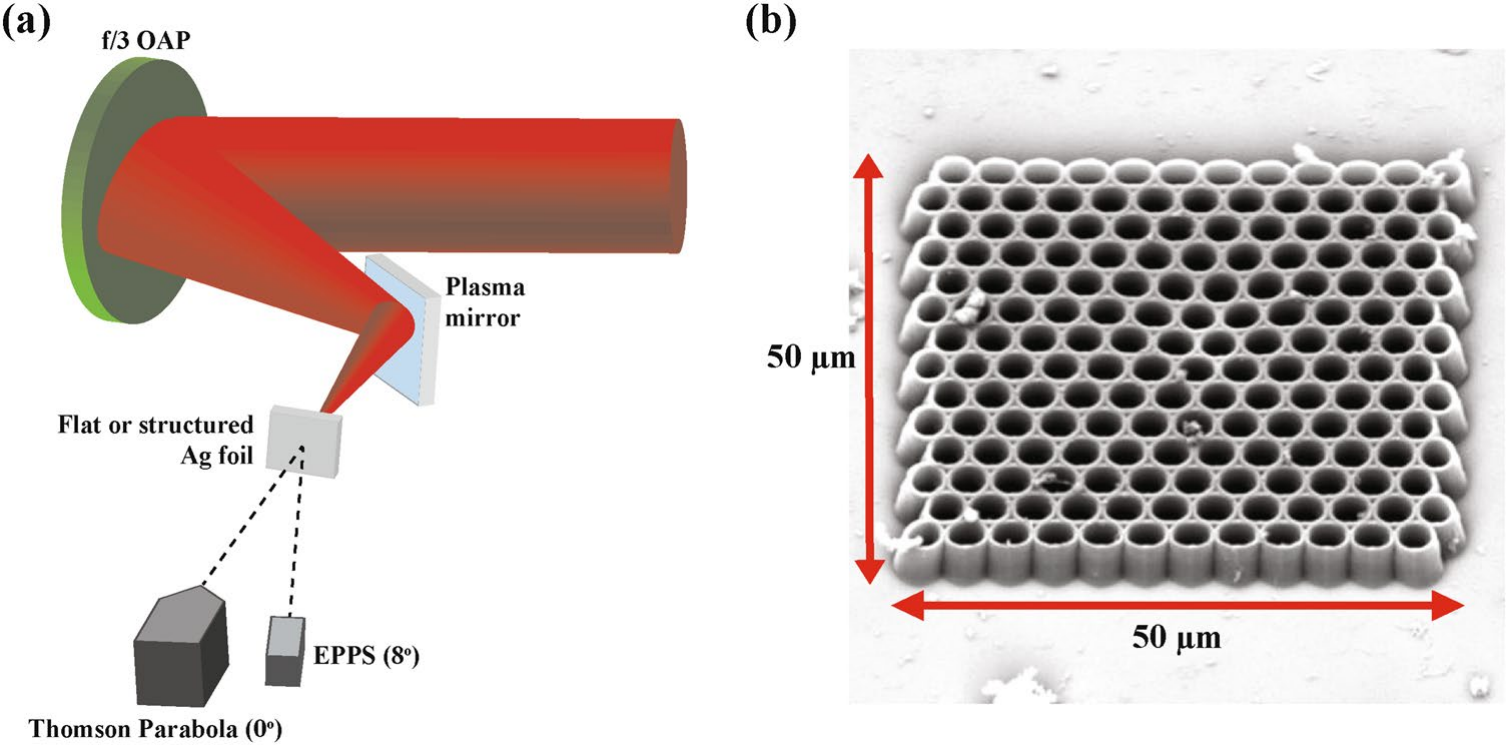
(**a**) Sketch of the experimental setup with the Texas Petawatt Laser, not to scale. After the plasma mirror, the laser is normally incident onto the front surface, either directly onto a 1 ${\upmu \hbox {m}}$ flat Ag foil, or the microtube array. A Thomson Parabola (TP) ion spectrometer is placed along with the target normal with the EPPS placed off-axis. For several shots on each configuration, a radiochromic film (RCF) stack was placed 4 cm from the target rear surface in order to capture the full proton beam. This diagnostic is not shown in the setup because it blocks the target line-of sight to the TP and EPPS. (**b**) Scanning electron microscopy (SEM) image of a $3 \times 5$ microtube array 3D printed on a 1 ${\upmu \hbox {m}}$ Ag foil. The large array size relative to the laser spot size eases alignment, and guarantees the laser will hit the array.

The experimental setup at the Texas Petawatt (TPW) laser, as illustrated in Fig. 1a, is as follows. A laser pulse of wavelength $\lambda _0 = 1.057$  ${\upmu \hbox {m}}$ reflects off an f/3 off-axis parabola, focusing down to a FWHM spot size of 5.6 ${\upmu \hbox {m}}$ on target after a plasma mirror. The plasma mirror is a borosilicate slab, with an anti-reflective coating optimized for laser normal incidence on target. The plasma mirror enhances the laser intensity contrast to $\sim$ 10$^{10}$ beyond 100 ps, essential to preserving the integrity of the plastic microtubes 3D-printed onto the 1 ${\upmu \hbox {m}}$ Ag foils. The microtubes (Fig. 1b) were fabricated via the 2-photon polymerization (2PP) technique, and have a 3 ${\upmu \hbox {m}}$ inner diameter and 5 ${\upmu \hbox {m}}$ height (shorthand “$3 \times 5$”), with a 0.35 ${\upmu \hbox {m}}$ wall thickness. A single tube dimension was chosen to guarantee good statistics for each set of laser parameters studied ($\sim$ 10 data samples for each spectrometer), and this particular dimension was shown to have an exemplary performance by previous studies of the authors^39^. To benchmark the performance of the microtubes, flat Ag foils were studied under identical laser conditions. To measure the proton energy spectra from the target rear, a Thomson Parabola (TP) ion spectrometer was fielded at $0^{\circ }$ from target normal. In addition, a Radiochromic film (RCF) stack was placed 4 cm from rear target normal for several shots on each configuration, allowing the study of proton beam enhancement closer to the target than is feasible with the higher-resolution Thomson parabola. The RCF stack recipe remained unchanged throughout the experiment, with the first 8 active layers composed of the HD-type due to its lower sensitivity, followed by 11 layers of the more sensitive EBT-type^42^. Nickel filters ranging from 127 ${\upmu \hbox {m}}$ to 1016 ${\upmu \hbox {m}}$ thick were placed between each layer, resulting in a final stack configuration sensitive to protons up to 70.5 MeV.

The forward hot electron spectra were sub-sampled from a magnetic spectrometer known as an EPPS (Electron, Proton, Positron Spectrometer)^43^. Both the EPPS and TP dispersed the charged particles onto imaging plates, which have an active layer sensitive to incident radiation. The imaging plates were scanned with a General Electric Typhoon FLA 7000 IP scanner. The scanner digitizes the image into units of photostimulated luminescence (PSL). Imaging plate sensitivity depends on the species and energy of the particles detected. Therefore, calibrations to absolute numbers of electrons^44^ and protons^45^ were applied during the data analysis.

## Results

### Experimental measurements

**Figure 2:**
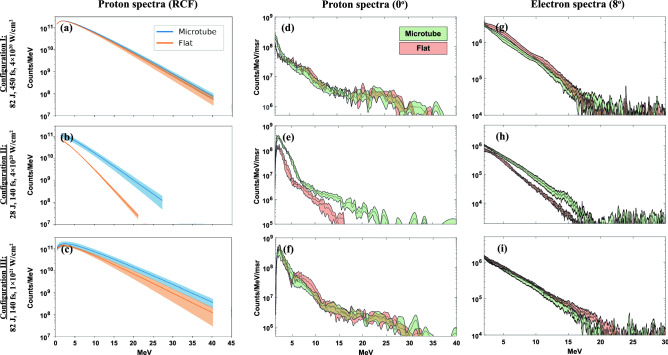
Proton and electron spectra for each laser configuration. (**a–c**) Maxwell–Boltzmann fits to the RCF spectra, with good correspondence to the Thomson parabola at $0^{\circ }$ (**d–f**). The full energy configurations (top and bottom rows) show a minimal difference between proton spectra from flat and microtube targets. The middle row, however, indicates that for the 28 J configuration, microtubes enhance maximum proton energy by 50%, and total proton yield by 3$\times$ (**b,e**). A corresponding enhancement in electron spectra, namely a 12% temperature increase, is also observed in (**h**). The width of each spectrum represents the error bands, as averaged over $\sim$ 3–5 shots for RCF, and $\sim$ 10 shots for the TP and EPPS. Key takeaways from the spectra are summarized in Table 2.

Figure 2 shows the proton and electron spectra from each laser configuration. For the RCF (Fig. 2a–c), the proton spectra shown are Maxwell–Boltzmann fits to the dose deposited on each layer of film. There is good agreement with the raw proton spectra from the TP, which affords more statistics ($\sim$ 10 shots each). Both full energy (82 J) configurations (top and bottom rows) performed similarly, regardless of pulse length and target type. Though the average microtube performance is higher, the error bands, representing the standard deviation, overlap on all diagnostics for the 82 J shots. This is not the case for Configuration II, which has the same intensity as Configuration I but the pulse compressed from 450 to 140 fs. To compensate, the energy was lowered to 28 J on target. For these laser parameters, the microtube targets outperformed flat foils in both proton and electron generation. Figure 2b,e both indicate that microtube targets increase the proton cutoff energy by $\sim$ 50%. The total yield of highly energetic protons (> 10 MeV) was also increased, by a factor of $\sim$ 3$\times$ on the Thomson parabola (TP) and $\sim$ 8$\times$ on the RCF stack. The TP could not detect the full effect of the enhancement because it cannot detect the entire forward-directed proton beam due to its small 300 ${\upmu \hbox {m}}$ pinhole. A comparison of relative maximum energy and proton yield is summarized in Table 2. The increase in yield also corresponds with an improvement in total conversion efficiency for laser to proton beam energy. For all protons > 1 MeV, microtubes increase the conversion efficiency from 0.55% to 1.87%. The other laser configurations perform almost identically regardless of target type, with an average conversion efficiency of 1.89% for Configuration I, and 1.84% for Configuration III. Conversion efficiencies on the order of a percent are typical of TNSA^46,47^, and can be increased with either a preplasma or a ps-laser pulse^48^, providing evidence that Configuration II is a high contrast laser–plasma interaction. The potential to produce this same enhancement in conversion efficiency with microtube targets (3.4$\times$) with the full energy laser (Configurations I & III) is elaborated upon in the Discussion.

The electron spectra from each laser configuration occupy a similar trend (Fig. 2g–i). The spectra from the full energy configurations have overlapping error bands, while the 28 J configuration shows a 12% increase in the average electron temperature $T_{hot}$. Microtube targets yielded an average temperature of 3.15 MeV ± 0.15 MeV, while the temperature from flat targets was 2.81 MeV ± 0.02 MeV. These temperature measurements were extracted via a least-squares regression, with excellent fits of $R^2 > 0.99$. This electron enhancement with microtube targets provides direct evidence that a pre-formed channel can improve the coupling efficiency of the laser energy to hot electrons.

**Table 2:** Summary of proton beam enhancement as measured by both ion diagnostics.

Config.	E$_{max,tube}$/E$_{max,flat}$ ($0^{\circ }$ TP)	E$_{max,tube}$/E$_{max,flat}$ (RCF)	Yield$_{tube}$/Yield$_{flat}$ ($0^{\circ }$ TP)	Yield$_{tube}$/Yield$_{flat}$ (RCF)
I	$\sim$ 1	$\sim$ 1	$\sim$1	$\sim$ 1
II	1.56	1.38	2.90	8.25
III	$\sim$ 1	$\sim$ 1	$\sim$ 1	$\sim$ 1

Due to overlap in the error bands of the proton spectra, there is no measurable difference in E$_{max}$ and relative proton yield for the full energy shots, Configurations I and III. Configuration II, with its short pulse length (140 fs) and lower energy (28 J), results in the best relative performance of microtube targets compared to flat foils. As determined by the averaged proton spectra, E$_{max}$ increases by $\sim$ 50%. For highly energetic protons > 10 MeV, the yield increases by $\sim$ 3$\times$ at $0^\circ$, and $\sim$ 8$\times$ across the entire captured beam.

### Simulations of the laser–plasma interaction

**Figure 3:**
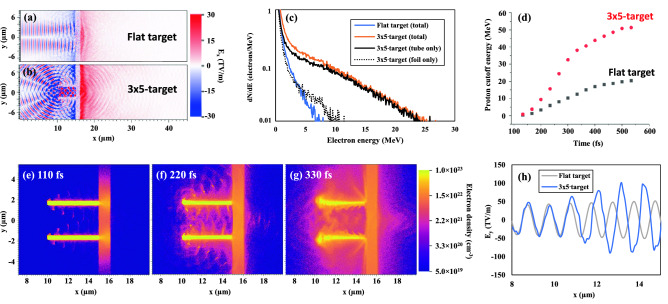
Comparison of flat and microtube ($3 \times 5$) targets for the optimum laser configuration (28 J, 140 fs). The longitudinal electric field in the flat foil (**a**) is outperformed by the microtube target (**b**), shown at approximately laser peak arrival at 233 fs. This stronger electric field is indicative of a dramatic increase in the acceleration of electrons from microtube targets (**c**). The distribution of forward-accelerated electrons from the foil is similar, regardless of whether a target structure is in place. Microtubes provide an additional source of hot electrons that dominates the energy spectrum. As the simulation progresses, the maximum proton energy doubles relative to flat targets (**d**). Evolution of extraction from the microtube walls, respectively at 110, 220, and 330 fs (**e–g**). The modulation of the incident laser field is shown for both target types at 266 fs (**h**).

To investigate the role of microtubes in enhancing electron and proton generation, a combination of both radiation-hydrodynamic (rad-hydro) and particle-in-cell (PIC) simulations are beneficial to best characterize the entire laser–target interaction. After the plasma mirror, the TPW laser is a high-contrast system ($\sim \,10^{10}$), yet the attenuated prepulse is still not eliminated. To determine the role of the laser prepulse, the rad-hydro code FLASH^49^ was executed for both the $4 \times 10^{20}$ W/cm$^2$ and $1 \times 10^{21}$ W/cm$^2$ laser pulses, as the intensity of the TPW prepulse depends on the main pulse peak intensity. For the lower intensity case of $4 \times 10^{20}$ W/cm$^2$, the prepulse produces an undercritical preplasma inside the microtube, whereas for the $1 \times 10^{21}$ W/cm$^2$ laser, the preplasma is overcritical. Figure 3 summarizes the results calculated in EPOCH^50^ for the optimum laser case. The longitudinal electric fields, which are responsible for accelerating the contaminant layer protons via the TNSA mechanism, are higher by a factor of 2$\times$ for the microtube case (Fig. 3a,b). This same enhancement factor was also observed in a similar numerical study by Snyder et al. for a higher intensity ($5 \times 10^{22}$ W/cm$^2$) laser pulse^51^. The electrons dragged out of the tube walls provide the primary source of enhancement^24,25^. Once inside the tube, the electrons can interact directly with the laser field, further increasing the maximum electron energy^52^.

With tube structures, the electron temperature increases by nearly a factor of 3$\times$, from 2.34 to 6.85 MeV. The structures also improve the laser coupling to the population of electrons originating from the flat substrate only, increasing this sub-sample of the electron temperature by 85%. However, electrons originating from the tube walls dominate the total accelerated population by orders of magnitude, as shown by the overlap of the black and orange lines in (c). These lines have near-identical temperatures, within 4% of each other, of $T_{hot} \approx 7$ MeV. All fits were extracted with the least-squares method, with coefficients $R^2 > 0.97$. The correlation between $E_x$ and $T_{hot}$ agrees with the TNSA scaling of $eE_{x} \sim T_{hot}/\lambda _D$^8^. As a result, as the simulation progresses, protons accelerated from the rear surface of microtube targets gain double the maximum energy relative to flat foils (Fig. 3d). To confirm the undercritical preplasma could be negated, a second simulation was run for these laser parameters, with the preplasma imported, to confirm there were no discernible differences. As a final result, including the preplasma in the simulation dampens the maximum proton energy by only $\sim$ 1 MeV.

The simulations also qualitatively support the experimental results that microtube targets enhance proton yield, with these advanced targets generating 3$\times$ more protons with energy > 10 MeV. Though this corresponds with the enhancement measured in the target normal direction by the Thomson parabola, it underestimates the total highly energetic proton yield from the beam. The $\sim$ 8$\times$ enhancement factor observed on the RCF suggests a discrepancy between the contaminant layers of the experiment and simulation. The contaminant layers that source the protons have proven difficult to characterize^53,54^, and estimates range from a few nm^55^ to 1 ${\upmu \hbox {m}}$^56^, varying significantly depending on material adhesion and environmental factors^57^.

The evolution of the electron density map Fig. 3e–g gives a qualitative picture of the tube’s role in providing electrons. 110 fs into the simulation, as the laser pulse is ramping up, periodic structures emerge from the tube walls (e). These structures of periodicity $\sim \lambda _0$ result from laser fields strong enough to disrupt the Langmuir oscillation within the dense plasma^24,58^. These features continue to grow as the laser peak reaches the target (f). As the laser pulse ramps down 330 fs into the simulation, the tube walls are largely disrupted, forming an overcritical channel (g). Before the channel becomes overcritical, however, the plasma expanding from the tube walls causes the laser to focus, doubling the laser field (h). For the peak intensity of the laser interaction around $\sim$ 200–300 fs, the narrowing channel (f) and doubling laser field (h) are consistent with the so-called “depletion regime” identified by Ji et al. in their 3D numerical study on lensing effects in microtubes^23^. In the depletion regime, electrons extracted from the tube walls can self-focus the laser pulse inside the tube. Lasers of intensities $10^{20}{-}10^{21}$ W/cm$^2$ fall into the heart of the depletion regime, meaning the focusing effect of the microtube can drop as low as a factor of $\sim$ 2$\times$ according to their numerical study. A 2$\times$ laser field amplification was observed here, corresponding to a 4$\times$ increase in intensity. This deviation is likely a result of the different tube geometry investigated (4.8 ${\upmu \hbox {m}} \times 120$ ${\upmu \hbox {m}}$), as well as the constraints of 2D simulations. Higher intensity laser pulses ($\gtrsim 10^{22}$ W/cm$^2$) are predicted to drive inward wall motion relativistically, compressing the transverse laser profile in a piston-like fashion. As this behavior is not evident from the electron density evolution Fig. 3e–g, lying in the depletion regime suggests that the lensing effect is only a minor contributor to enhancing proton acceleration.

**Figure 4:**
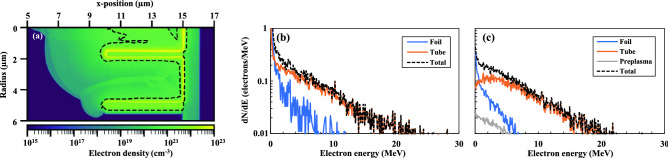
The role of the preplasma for structured targets at high drive energy (82 J). (**a**) FLASH simulations produced a density profile generated by the prepulse, with a contour (dashed line) indicating the overcritical region. These data were extracted 3 ps before the peak of the main pulse arrived. (**b,c**) Compare the spectra of forward-accelerated electrons without and with the preplasma, respectively, from each target component. Tube structures are the dominant source of hot electrons. The over-critical preplasma in (**a**) reduces the total number of electrons accelerated > 100 keV by $\sim$ 50% (**c**). The density profile of (**a**) was assumed to be cylindrically symmetric for the EPOCH simulation.

The intensity profile of the laser prepulse depends on that of the main pulse. Consequently, whether a fixed intensity pulse is stretched to 140 fs or 450 fs, it will interact with a preplasma of the same density profile. The FLASH code predicts that for the 10$^{21}$ W/cm$^2$ laser, the preplasma is overcritical and causes the central microtube to behave like a closed shutter (Fig. 4a). EPOCH simulations, importing this overdense preplasma profile, were executed for a main pulse of 140 fs, 10$^{21}$ W/cm$^2$, corresponding to Configuration III of the experiment. With this laser pulse, two cases were studied in EPOCH to compare the role of the preplasma inside the microtube for high-energy pulses. Without the preplasma (Fig. 4b), electrons sourced from the microtube dominate the hot electron population. This behavior is similar to the optimum case, where the pulse length is the same but the energy is attenuated to 28 J (Fig. 3c). Though this preplasma does not change this qualitative behavior, the number of accelerated electrons is dramatically reduced. The energy spectra of Fig. 4c show that the electron counts from the tube (orange line) break from an exponential spectrum below 7 MeV. Integrating over the total electron counts (black lines), the preplasma decreases the number of accelerated electrons > 100 keV by $\sim$ 50%. Though the preplasma provides an additional source of electrons (grey line), those electron counts are nearly two orders of magnitude too small to contribute on the same scale as the tubes. In effect, the simulations indicate that the highest intensity laser pulse is interacting with an overcritical plasma slab of 6 ${\upmu \hbox {m}}$ in thickness. Similar performance was observed experimentally for the 450 fs, $4 \times 10^{20}$ W/cm$^2$ laser pulse, which also contains 82 J of energy. Though it interacts with an undercritical preplasma, such long pulse lengths have been shown to shutter the tube before the peak of the interaction, as studied in detail by Bailly-Grandvaux et al.^39^.

## Discussion

The laser-acceleration of protons from microtube targets was investigated experimentally for three laser configurations, shedding light on the interplay of laser intensity, pulse duration, and energy. For the laser parameters studied (Table 1), it was found that from the low energy laser case (28 J), microtube targets outperformed flat foils in maximum proton energy by 50%, and highly energetic proton yield by more than 8$\times$. When the laser was at full energy (82 J), the proton energy and yield showed no statistically significant dependence on pulse duration (140 fs or 450 fs), intensity ($4 \times 10^{20}$ W/cm$^2$ or $1 \times 10^{21}$ W/cm$^2$), or target type. FLASH simulations indicate that despite the presence of a plasma mirror, the highest intensity laser case drives an overcritical preplasma, shuttering the microtube from the main pulse. For the longer pulse case (450 fs at $4 \times 10^{20}$ W/cm$^2$), microtube performance is also limited, as the microtube is shuttered during the main pulse interaction^39^. For intensities of $4 \times 10^{20}$ W/cm$^2$, the preplasma is low enough density to be classically transparent. According to the simulations, for a sufficiently short pulse (140 fs), the sheath field and electron temperature are more than doubled, as is the proton cutoff energy. Though the trend is in qualitative agreement with the experimental results, it is well established that 2D PIC simulations overestimate the experimental electron temperature and cutoff energy enhancement^59^, which in this case are 12% and 50%, respectively.

The simulations were also not able to capture the $\sim$8 $\times$ increase in proton yield, likely because the contaminant layer is difficult to characterize. The main source of proton enhancement is driven by additional hot electrons accelerated from the tube walls, while light intensification is a minor contributor. For the highest intensity laser ($I = 1 \times 10^{21}$ W/cm$^2$, 140 fs, 82 J), including the preplasma decreases the number of hot electrons > 100 keV by 50%. The other high-energy laser configuration, with its pulse stretched to 450 fs, was not simulated as the tube shuttering effect at $\sim$ 500 fs pulse lengths was studied extensively in Bailly-Grandvaux et al.^39^. The experimental results presented here are consistent with this reference, and support that optimizing the microtube geometry for these pulse lengths is a topic of future study. Though performance with the compressed pulse and higher preplasma is comparable, with conversion efficiencies of $\sim$ 2%, the main pulse interacting with a preplasma is associated with a larger shot-to-shot variation due to the hydrodynamic times scales of the expanding preplasma^60^. Therefore, optimization with a high contrast laser pulse is preferred due to its ion source stability. In addition, a high contrast interaction at arbitrarily higher intensities should continue the factor of $\sim$ 3$\times$ conversion efficiency enhancement from the microtube targets.

For sufficiently high-intensity laser pulses $\gtrsim 10^{20}$ W/cm$^2$, numerical and experimental results on structured target enhancement begin to diverge. For a variety of numerical studies on laser-driven radiation sources^23,24,61^, microstructured targets of a fixed geometry consistently outperform flat targets for arbitrarily high laser intensities. Approximating a high contrast laser pulse to be of infinite contrast may hold for lasers $\lesssim 10^{20}$ W/cm$^2$, but in reality, higher intensity pulses are accompanied by increasingly damaging prepulses. The experimental study presented here suggests that for higher intensity laser pulses, the optimum microstructure geometry should be modified to continue the trend of superior performance of structured targets, such as the > 3$\times$ enhancement in conversion efficiency observed in Configuration II. Future experiments are of interest to determine the modified target geometries necessary as high contrast lasers with intensities beyond $10^{22}$ W/cm$^2$ become more widely available. With an optimized geometry, such high-intensity lasers are predicted to strongly self-focus within the microtube via a relativistic collapse of the channel radius^23^. Via a laser intensity scan, a transition into the self-focusing regime should leave the experimental signatures of improved electron heating and increased proton cutoff energy. To reach the intensities required for this experiment, next-generation facilities, such as those at the ELI and APOLLON installations, operate with ultrashort (10s of fs) pulses. Experimentally studying microtubes at these pulse durations will further broaden the map of microtube performance across the laser parameter space.

Many applications benefit as structured targets become further optimized. As demonstrated in this work, under the right conditions, microtube targets can dramatically increase proton production. This energy and yield enhancement is of great utility for dense plasma physics. For example, larger fluxes provide more protons per energy bin, which is advantageous when injecting a proton beam into an energy selector. This shows promise for laser-driven injectors for radiofrequency accelerators^62^, as well as providing higher resolution data-sets for warm dense matter stopping power studies^4^. Higher proton fluxes also directly benefit neutron generation for nuclear science and national security applications, as energetic protons can be converted into neutrons via a “catcher” material, such as lithium or beryllium^63,64^.

The MeV electrons from laser–solid interactions can also produce intense sources of MeV photons, or $\gamma$-rays, when injected into a high-Z substrate^65,66^. Increasing the electron population via microtube targets is a promising avenue toward increasing the conversion efficiency of $\gamma$-ray sources, which has been predicted through simulations for microtubes^23^ and observed experimentally for nanowire targets^17^. Recent progress in target fabrication includes microtube targets filled with relativistically transparent foams, which have already been shown to improve the generation of hot electrons and betatron photons relative to flat targets^67^. A study directly comparing proton, electron, and $\gamma$-ray generation from hollow and foam-filled microtubes is a topic of future investigation, both in the intensity regime studied here and beyond.

## Methods

### Simulation conditions

To evaluate the role of a tube pre-expansion by the laser prepulse on-target performance, we performed 2D FLASH radiation hydrodynamic simulation of a microtube target. The microtubes have an inner diameter of 3 $\upmu$m and a height of 5 $\upmu$m and are attached on a 1 ${\upmu \hbox {m}}$ thick Ag foil. The simulations are performed in 2D cylindrical geometry, using the equation-of-state and multi-group opacity tables from PROPACEOS^68^. The prepulse intensity pulse shape is calculated from the laser intensity contrast measured by the facility with a third-order autocorrelator. The reflectance of the plasma mirror anti-reflective coating is calculated using manufacturer curves and has been evaluated to 0.07$\%$ with the s-polarized laser at $\lambda _0 = 1.057$ ${\upmu \hbox {m}}$ and with an incidence angle of 30$^{\circ }$ on the plasma mirror. FLASH uses an Adaptative Mesh Refinement (AMR) and the coarsest/finest mesh size used is 0.1 ${\upmu \hbox {m}}$/0.006 ${\upmu \hbox {m}}$ in both directions, respectively. The time step is constrained by a Courant–Friedrich–Lewy (CFL) limit of 0.4. The laser deposition is realized through a 3D ray-tracing projection on the cylindrical domain, and the laser spatial profile is Gaussian with an FWHM spot diameter of 5.6 ${\upmu \hbox {m}}$ and is focused onto the foil’s front surface at (r,z)=(0, 0).

The density profile created during the FLASH simulation was then imported into EPOCH. The simulation domain is of the size ($L_x,L_y$) = (15.0, 45.0) ${\upmu \hbox {m}}$ , where *x* is the laser propagation direction, and *y* is the transverse direction in which the laser electric field is polarized. The number of computational cells is ($N_x, N_y$) = (4500, 1600), corresponding to a cell size of $\Delta x = \Delta y = 0.01$ ${\upmu \hbox {m}}$. At the left-hand boundary of the 1 ${\upmu \hbox {m}}$ Ag foil ($x=15$) ${\upmu \hbox {m}}$, where the 1.057 ${\upmu \hbox {m}}$ wavelength laser, injected from the left, focuses to a FWHM spot size of 5.6 ${\upmu \hbox {m}}$. The Ag foil is initialized with ionization Ag$^{30+}$, at a solid density of $60n_c$, with 400 particles/cell. The CH microtubes are fully ionized with a density of $46n_c$, at 100 particles/cell. The rear surface has a 60 nm thick layer of H contaminants, with a density of 3$n_c$ and 400 particles/cell. The laser and microtube are both centered in *y* of the simulation box. The microtube is of 3 ${\upmu \hbox {m}}$ inner diameter, 5 ${\upmu \hbox {m}}$ in height, and has walls 350 nm thick, corresponding to physical measurements of the microtube targets. The laser intensity, energy, and pulse duration were varied, corresponding to the parameters of Table 1, for both microtube and flat targets.

### Advanced target fabrication

Microtubes on Ag foil targets were fabricated via a multistep approach, reliant on the additive manufacturing technology known as 2 photon polymerization (2PP). First, 300 ${\upmu \hbox {m}}$ thick silicon washers with a 1 mm inner diameter and 3 mm outer diameter were laser machined, then glued to a 1 micron thick Ag foil. Individual washer and foil assemblies were then separated using laser machining for mounting on adhesive Gel-Pak substrates. The raw materials for 2PP were then prepared by mixing the acrylate monomers, Dipentaerythritol Penta/Hexa Acrylate, and Bisphenol A ethoxylate diacrylate, in a 40:60 mass ratio. This ratio was carefully chosen, so the index of refraction of the monomer blend matches that of the objective lens of the laser required to drive 2PP^69^. Subsequently, a sensitive 2PP initiator^70^ was dissolved at a concentration of 0.2% by mass. This photosensitive monomer mixture was dispensed on top of the foil assembly and placed in a custom 2PP system.

The main components of the system include a Ti:sapphire femtosecond pulsed laser, a high-speed shutter, and an oil immersion objective for focusing the laser, all with a high degree of axial control. The oil immersion objective is immersed directly into the monomer solution, in which the Ag substrate and washer sit at the bottom. Microtubes are fabricated, directly onto the Ag substrate, via laser scanning multiple layers of hexagonally close-packed arrays of circles. The resolution of the 2PP print process is submicron, allowing for fabrication of the $3\times 5$ tubes. Uncured monomer is removed via several rinses in ethanol and then the microtube array is dried in air. After drying, $\sim$ 20 ${\upmu \hbox {m}}$ holes were laser-drilled in the Ag foil above and below the microtube array to ease target alignment during the experiment. After the batch was completed, a sacrificial target was imaged via scanning electron microscopy to confirm the target parameters (microscopy image shown in Fig. 1b).

## Data Availability

The datasets generated during and/or analysed during the current study are available from the corresponding author upon reasonable request.
